# Gene expression analysis for feed efficiency trait in liver tissue of lactating Girolando cows

**DOI:** 10.1590/1678-4685-GMB-2025-0036

**Published:** 2026-01-19

**Authors:** Daniele Ribeiro de Lima Reis Faza, Mariana Magalhães Campos, Thierry Ribeiro Tomich, Fernanda Samarini Machado, Luiz Gustavo Ribeiro Pereira, Robert Domingues, Ana Luiza Franco, Marta Fonseca Martins, João Cláudio do Carmo Panetto, Marcos Vinicius Gualberto Barbosa da Silva, Wanessa Araújo Carvalho, Marco Antonio Machado

**Affiliations:** 1 Embrapa Gado de Leite, Juiz de Fora, MG, Brazil.; 2 Universidade Federal de Juiz de Fora, Juiz de Fora, MG, Brazil.

**Keywords:** Residual feed intake, dairy cattle, RNA-seq, Girolando

## Abstract

The selection of high feed efficiency (FE) animals impacts sustainability and profitability of beef and dairy cattle production systems. An approach to investigate the mechanisms of FE involves analyzing gene expression profile in liver. This study used residual feed intake as a metric of FE to select 10 Gir x Holstein crossbred cows (Girolando F1) divided into high (HE) and low (LE) FE groups. Hepatic biopsies were used for differential gene expression investigation using RNA-seq analyses which revealed 20,787 known genes mapped accordingly to the bovine reference genome. The comparison of HE and LE revealed 149 significantly differentially expressed genes (DEG), 41 up-regulated, and 108 down-regulated in the LE group. Among DEG, some stood out as potential candidate genes, including DLK1, CACNG4, SLC2A12, SLC26A4, DUOX2, and DUOXA2. Functional enrichment analyses showed pathways that potentially influence FE, such as the negative regulation of leukocyte migration, regulation of calcium channel activity, negative regulation of cell migration and adhesion, extracellular matrix (ECM) organization, and thyroid hormone synthesis. ECM composition and immune system roles were also highlighted. These results could help understanding the mechanisms related to FE in dairy cattle and the development of selection strategies to improve this trait.

## Introduction

Global demand for animal-based food products is expected to increase by 20% until 2050. As an essential source of nutrients, meat, eggs, and dairy products will play a crucial role in ensuring food security, improving nutrition, and maintaining healthy diets (FAO, 2023). Producers face difficulties that reduce profit margins, such as labor shortage in the field, rising wages and input costs (Llanos et al., 2018). Consumers are increasingly demanding information about food safety, animal welfare, and the reduction of environmental impacts. Increasing livestock productivity appears to be one of the best ways to increase food production while reducing production costs and environmental impact (Vandehaar et al., 2016).

Brazil is a major global milk producer and has been showing growth in productivity, although it is still far behind other countries. The average milk yield in Brazil was 2,192 liters/milked cow/year while in the United States, the country with the highest productivity, this value exceeded 10,000 liters/milked cow/year (Embrapa, 2022). Girolando, which is the cross between Gir and Holstein, is the most used breed in milk production systems and it is also the fastest growing breed in semen sales in Brazil. This breed is widely used in the country, and approximately 80% of the milk production comes from Girolando animals. This crossbred can maintain a profitable level of production in different management systems and harsh climatic conditions and it has been gaining increasing domestic and international recognition and becoming the preferred breed for milk production in tropical areas (Silva et al., 2023).

Selecting animals that are more efficient in using the feed they ingest is a smart strategy to increase milk productivity in cattle production systems. Feed efficiency (FE) in dairy cattle can be defined as the efficiency in converting feed nutrients into milk and body weight (Madilindi et al., 2022). Residual feed intake (RFI) is the most important metric for evaluating FE, as it allows the identification of most efficiently and desired phenotypes. In lactating cows, RFI is estimated by the difference between the actual feed intake in dry matter or energy, measured over a long or several short-controlled periods, and the expected feed intake (Herd & Arthur, 2009). In Brazil, selection for feed efficiency (FE) has only been addressed in the recent years and most of the research is restricted to beef cattle. In the literature related to FE, most studies were done using animals from pure breeds specialized in milk production, adapted to temperate climate conditions, and majority of them were carried out with Holstein cows. Thus, there are few scientific reports on FE in other breeds, even European ones, such as Jersey and Nordic Red Dairy (Liinamo et al., 2015). Therefore, studying FE and understanding the genetic mechanisms of this in a synthetic breed (*Bos taurus* × *Bos indicus*), such as the Girolando breed, subjected to a tropical climate, represents a very relevant research theme.

Due to the role of the liver in regulating homeostasis, the immune system and especially in the metabolism and use of nutrients from food, such as carbohydrates, lipids, proteins, minerals, and vitamins essential for milk production, the hypothesis of this work is that a differential expression of genes in the liver tissue of lactating Girolando F1 cows could be detected between animals showing different levels of FE.

Thus, the objective of this work was to evaluate the expression of genes related to FE in the liver tissue of primiparous Girolando F1 cows displaying contrasting RFI.

## Material and Methods

### Sampled animals

Experiment trials were conducted at the Multi-user Livestock Bio efficiency and Sustainability Laboratory, located at the José Henrique Bruschi Experimental Field of Embrapa Dairy Cattle, located in Coronel Pacheco, Minas Gerais, Brazil. Using the same population evaluated in a previous experiment conducted by Sacramento et al. (2024), a total of 29 primiparous Girolando F1 cows were randomly selected for evaluation of residual feed intake (RFI). The experiment started with animals showing an initial body weight averaging 563 ± 40.1 kg and were 2.5 ± 0.09 years old. The experiment was conducted in free-stall conditions and the cows were fed *ad libitum* with a total diet containing corn silage, Tifton hay and corn-soybean-based concentrate. The ratio between roughage and concentrate and the chemical composition of the diets changed accordingly to the days in lactation. Weight gain, morphological measurements, milk production, water consumption, and feed intake were daily assessed. Electronic troughs (AF-1000, Intergado^®^ Ltd., Contagem, Brazil) and automatic drinkers with a body weighing platform (VW-1000, Intergado^®^ Ltd., Contagem, Brazil) were used to evaluate feed intake and animal weight. Milk production was recorded daily by automatic electronic meters coupled to the milking system (DeLaval, HB30, Tumba, Sweden). The experiment covered the whole lactation period of 300 days and cows remained non-pregnant during the study. Health and clinical parameters were daily monitored. 

From the 29 cows evaluated, a total of 10 animals showing extreme RFI values of the normal curve were selected to maximize the contrast between experimental groups and to facilitate the identification of candidate genes for FE: five high efficiency (HE) and five low efficiency (LE) animals. The HE group showed an average RFI of -0.956 ± 0.302 kg/d and the LE group showed an average RFI of 0.646 ± 0.192 kg/d. The groups were statistically different from each other by the t-test (p<0.0001).

### Liver tissue biopsies 

A total of 5 mL of 2% lidocaine anesthetic was injected under the skin and intercostal muscles at the insertion site of the biopsy instrument. Fifteen to 30 minutes after injection, a semi-automatic biomedical soft tissue biopsy cannula (14G x 200 mm) was inserted. Approximately 10 to 20 mg of liver tissue were collected and immersed in a solution of RNAlater^®^ (Ambion, Austin, Texas, USA) and stored at 4 °C for 24 h. After 24 h, the samples in RNAlater^®^ were stored at -20 °C until RNA extraction. Biopsies collection was aseptic and performed by a veterinarian on the 100^th^ day of lactation, which is the peak of lactation in dairy cows. 

### RNA extraction and sequencing 

Total RNA was extracted from liver tissue biopsies using the QIAGEN TissueRuptor mechanical grinder and the RNeasy^®^ Mini Kit (QIAGEN, Hilden, Germany) following a DNase enzyme treatment step under manufacturer’s guidelines. The quantity and quality of the extracted RNA were assessed using the NanoDrop® ND-1000 spectrophotometer (NanoDrop Technologies, Wilmington, DE, USA) and the Agilent 2100 Bioanalyzer microfluidic capillary electrophoresis instrument (Agilent Technologies, Foster City, CA, USA). RNA Integrity Number (RIN) value above 7.0 was the parameter used to determine the RNA integrity threshold. The mRNA sequencing was performed using the Illumina NovaSeq 6000 platform (San Diego, CA), using 2x150bp paired-end reads, which generated approximately 30 million sequences, totaling approximately 9 giga bases (Gb) per sample. The sequence data was deposited at the Sequence Read Archive of the NCBI under the accession number PRJNA1330216 (https://www.ncbi.nlm.nih.gov/sra/PRJNA1330216) and will be available to download on 2026-10-01.

### Data analyses

Quality control of the mRNA sequence reads was performed using the programs FastQC Read Quality reports version 0.11.9 (Andrews, 2010), and fastp version 0.23.2 (Chen et al., 2018). After checking the quality of the reads, the sample trimming step was performed using the Trimmomatic software version 0.38 (Bolger et al., 2014), excluding adapter sequences, small sequences (<100 bp), low-quality sequences (Phred˂20) and unpaired sequences. The alignment of the paired reads was performed from the bovine reference genome ARS-UCD1.3, RefSeq annotation GCF_002263795.2, using the HISAT2 Version 2.2.1 tool (Kim et al., 2015). The samtools program version 1.3 (Li et al., 2009) was used to transform the file format and order the reads. Read counting was performed using featureCounts version 1.5.2 (Liao et al., 2014) and differentially expressed genes (DEG) were identified using the DESeq2 version 1.40.2package (Love et al., 2014) in RStudio version 4.3.1 (R Core Team, 2023). The criteria for considering statistically significant differentially expressed genes were p-value < 0.01 and log2FoldChange > 1 for upregulated genes or < -1 for downregulated genes. Using the Enhancedvolcano and heatmap3 packages in the RStudio environment, a Volcano plot was created highlighting all DEG, and a heat map for the most significant DEG (p-adj < 0.05). For pathway enrichment and protein-protein interaction (PPI) network analyses, the significantly upregulated and downregulated genes were analysed together using the STRING database, version 12.0 (Szklarczyk et al., 2023). The names of the DEG were entered as input in the name list, and *Bos taurus* was chosen as the organism for analysis. Each interaction is associated with a reliability score, which can assume values between 0 and 1. The closer to 1 the score, the greater the certainty of the existence of the interaction. The analysis parameters were defined for the complete STRING network, where the edges indicate functional and physical associations of proteins, with a minimum required interaction score of 0.4 (medium confidence). Disconnected nodes were hidden from the network. The enrichment analyses included Gene Ontology (GO: biological process, molecular function, and cellular component) and KEGG pathways. The statistical test used was the hypergeometric included in STRING, and Benjamini-Hochberg correction was applied for multiple testing. Enriched terms with a false discovery rate (FDR) < 0.05 were considered significant.

The experimental procedures with animals were approved by the Embrapa Gado de Leite Animal Use Ethics Committee under the registration number CEUA nº 926422031.

## Results

The RNA samples showed appropriate quantity and quality to be to sequenced. The average concentration of the RNA samples was 268.9 ng/uL ± 161.5 ng/uL and the average RNA Integrity Number (RIN) value of the samples was 7.3 ± 0.4. The sequencing procedure generated, on average, 74,967,988 reads and 11.4 Gb per sample of raw data. The CG content of all HE and LE animals ranged from 51.0 to 52.4%. On average, 97.34% of the read bases showed high quality with Phred Score values above 20, with 99% certainty that the read base was the correct base.

**Figure 1 -:**
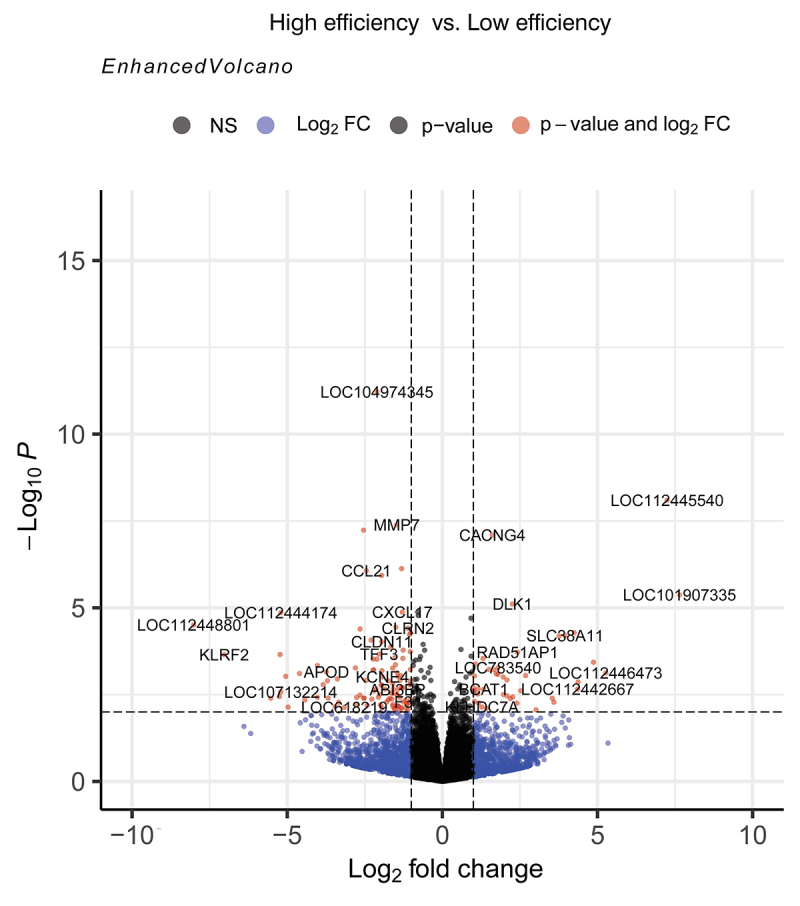
Volcano plot displaying the differentially expressed genes (DEG) found by RNA-Seq analyses in the liver of lactating Girolando F1 cows showing contrasting feed efficiency phenotypes: high feed efficiency (HE) × low feed efficiency (LE). Red dots indicate DEG with p-value <0.01 and log2FC > 1 or < -1, found in LE group. Down-regulated genes are shown on the left side and up-regulated genes are shown on the right side.

After trimming, mapping, and read-counting steps, an average of 56,121,969 reads were obtained per sample. Of the existing 30,543 genes in the bovine reference genome annotation ARS-UCD1.3, 20,787 genes were found in the analyzed samples. The differential gene expression analysis between high efficiency (HE) and low efficiency (LE) groups identified a total of 149 DEG in which 108 were downregulated and 41 were upregulated (Table S1). A volcano plot which graphically shows the DEG between HE x LE groups was generated (Figure 1).

**Figure 2 -:**
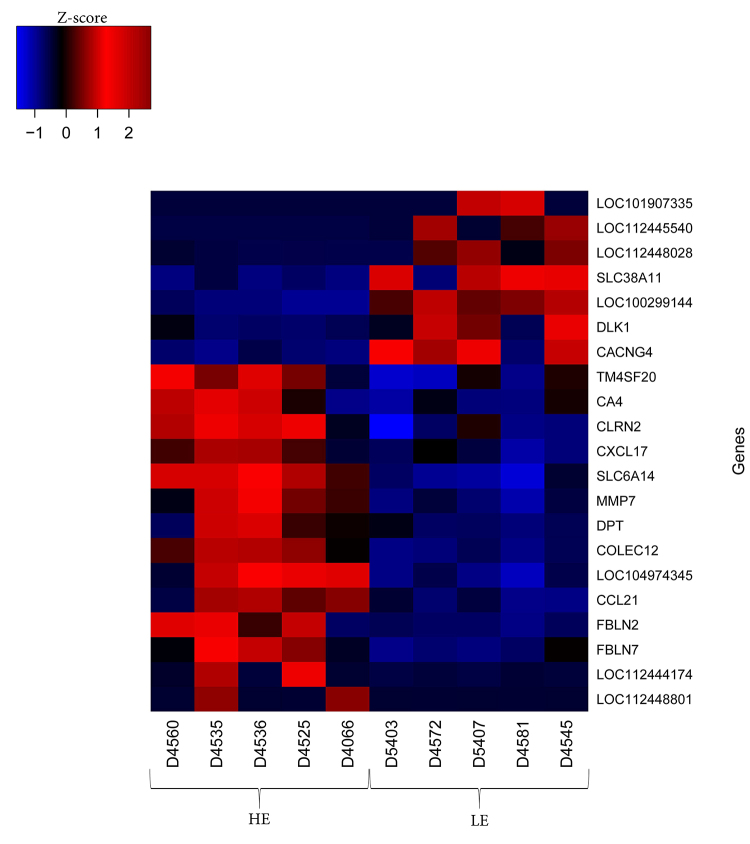
Heat map chart indicating the Z-score of 21 differentially expressed genes (p-adj <0.05) found by RNA-Seq analyses in the liver of lactating Girolando F1 cows showing contrasting feed efficiency phenotypes (HE and LE). The positive z-score indicates that the gene is over expressed, showed in red, while a negative z-score indicates that the gene is under expressed, showed in blue.

A heatmap with 21 DEG showing the highest significance (p-adj<0.05) was generated (Figure 2). The expression of these genes highlights the difference between the HE and LE groups. The *LOC101907335*, *LOC112445540*, *LOC112448028*, *SLC38A11*, *LOC100299144*, *DLK1*, and *CACNG4* genes were upregulated and *TM4SF20*, *CA4*, *CLRN2*, *CXCL17*, *SLC6A14*, *MMP7*, *DPT*, *COLEC12*, *LOC104974345*, *CCL21*, *FBLN2*, *FBLN7*, *LOC112444174*, *LOC112448801* were downregulated.

**Figure 3 -:**
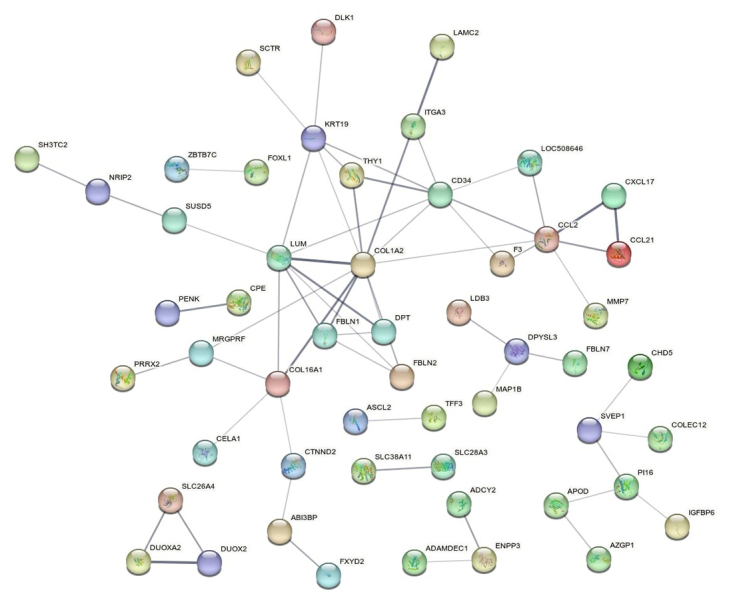
Protein interaction network from differentially expressed genes found by RNA-Seq in the liver of lactating Girolando F1 cows showing contrasting feed efficiency phenotypes.

The 149 DEGs between HE and LE groups were used to generate the protein interaction networks and functional enrichment by gene ontology analyses which are showed in Figure 3 and Table S2. The protein network exhibited 53 nodes, which denote proteins, and 62 edges, which denote interactions among proteins. The PPI enrichment p-value was 1.55E-13, which means that proteins show more interactions with each other than what would be expected for a random set of proteins of the same size and degree of distribution in the genome. The thickness of the edge line connecting the protein nodes in the network indicates the confidence of the data. Such enrichment indicates that the proteins are at least partially biologically linked to a network cluster. A total of nine clusters were found and the number of nodes in each cluster ranged from two to 28. 

## Discussion

In this study, the analyses were conducted based on a direct comparison between two groups (HE and LE). Therefore, all interpretations refer to the direct comparison between the HE versus LE groups.

The *DLK1* gene is a candidate gene for FE since it is associated with lipid metabolism in cattle and encodes a type 1 membrane glycoprotein. This gene is involved in adipogenesis, which may affect meat quality in production animals and functions as a negative regulator of adipocyte differentiation (Wang et al., 2020). Moon et al. (2002) found that mice lacking the *DLK1* gene showed obesity, increased serum lipid metabolites, skeletal malformation, and growth retardation. These results indicated that *DLK1* could play an essential role in adipocyte differentiation in mice. Albrecht et al. (2015) verified a higher expression level of the *DLK1* gene in the less marbled muscle of Holstein steers and that the number of *DLK1*-positive cells by immunohistochemistry was negatively associated with fat content. The authors suggest that *DLK1* is involved in the deposition and distribution of fat throughout body fat depots. In addition, two SNPs of the *DLK1* gene have been identified and shown to be associated with carcass and meat quality traits in Chinese Simmental steers. These results indicated that the bovine *DLK1* gene may affect lipid metabolism, and two SNPs could be applied as molecular markers for beef cattle selection in the future (Wang *et al.*, 2020).

The *CACNG4* gene encodes the auxiliary subunit gamma 4 of the voltage-gated calcium channel, allowing the entry of Ca+2 into the cell, which in turn uses it as a secondary messenger in cell functions and differentiation. Therefore, these aspects of *CACNG4*, indicated that it is related to information transmission activities and nerve cell formation (Yin et al., 2016). Voltage-gated calcium channels play an important role in both the nervous and cardiovascular systems (Paredes-Sánchez et al., 2020). Recent studies associated single nucleotide polymorphisms (SNP) variations in the intronic part of *CACNG4* gene with temperament traits in Brahman cattle (Paredes-Sánchez *et al.*, 2020; Ruiz-de-la-Cruz et al., 2023). In this latter work, the SNP Rs3423464051:G>A in the *CACNG4* gene was associated with exit speed and temperament traits. The authors stated that *CACNG4* is a candidate gene that requires more detailed analyses to reveal its role in temperament-related traits. In this sense, bovine temperament could be also related to feed efficiency, since more stressed animals expend more energy. In the gene ontology analysis, the regulation of cation channel activity was one of the enriched biological processes which included *CACNG4*.

In this study, five genes encoding proteins of the solute carrier group were differentially expressed. Solute transporters (SLCs) are the largest group of transporters, including transporters of inorganic ions, amino acids, neurotransmitters, sugars, purines, and fatty acids, among other substrates. *SLC38A11*, the only gene of the SLC family that is overexpressed in the contrast of HE x LE groups, belongs to the solute carrier family 38 (*SLC38*) and is, together with the *SLC32* and *SLC36* families, the only known member of the amino acid/polyamine/organocation transporter superfamily, also called the β group. On the other hand, four other genes of the SLC family are less expressed: *SLC6A14*, *SLC2A12*, *SLC28A3*, and *SLC26A4*. The *SLC6A14* gene encodes a transporter of neutral and cationic amino acids. The link between *SLC6A14* and obesity was investigated in wild-type and *SLC6A14* −/− mice. On a high-fat diet, *SLC6A14* −/− mice gained more weight than wild-type mice and also developed fatty liver and metabolic syndrome. In humans, a SNP in the 3′-untranslated region (3′-UTR) of the *SLC6A14* gene is associated with obesity (Sivaprakasam et al., 2021).

The *SLC2A12* gene encodes the GLUT12 protein, one of the membrane proteins responsible for the passive transport of glucose into the cell. The bovine GLUT family of glucose transporters comprises 12 proteins, which play a crucial role in cellular metabolism determined by their substrate specificity, expression in different tissues (organs) and the physiological state of the animal (Ostrowska et al., 2015). Glucose transporters (GTs) play a fundamental role in glucose homeostasis in livestock species, interfering in energy metabolism and, therefore, in the capacity to produce meat or milk. During lactation, the mammary gland uses large amounts of glucose, mainly for the synthesis of lactose, which is transported to the mammary epithelial cells by GTs. Changes in the expression of GT genes can lead to changes in the concentration of glucose transport proteins, which in turn, can alter the glucose supply to animal tissues and organs, such as the liver and mammary gland, and thus influence metabolism in general (Zwierzchowski et al., 2023). These authors also reported the presence of a SNP in the 5’ promoter region of the *SLC2A12* gene in Holstein cattle and correlated this SNP with increased milk production. According to the authors, the SNP g.-671C > G (NC_037336.1: g.72224078C > G) may be an effective molecular marker for cattle production traits and that the CC and CG genotypes are associated with higher productivity. Furthermore, qPCR analyses of cows with the CC genotype demonstrated a relative abundance of *SLC2A12* mRNA three times higher than that of CG cows in somatic cells freshly isolated from milk. These findings corroborate our results, in which HE lactating cows expressed more SLC2A12 than LE lactating cows, which may indicate different levels of GLUT12 on the membrane surface, influencing glucose transport.

The transporters encoded by the *SLC28A3* gene (Table S1) are Na+-dependent, belong to the nucleoside transport family and are involved in the transport of nucleosides, such as adenosine and cytidine, required for DNA and RNA synthesis, across the cell membrane. They regulate multiple cellular processes, including neurotransmission, vascular tone, adenosine concentration near cell surface receptors, and transport and metabolism of synthetic nucleoside analogue drugs (Ritzel et al., 2001).

*SLC26A4* is the gene that encodes the glycoprotein pendrin, which is expressed in several tissues, such as the inner ear, thyroid, liver, and airways. Pendrin is an anion exchanger responsible for the efflux of iodide ions (I-) in thyrocytes and for mediating the exchange of chloride/bicarbonate ions (Cl− /HCO−3) (Rebeh et al., 2010). The main function of the thyroid is the production of triiodothyronine (T3) and tetraiodothyronine or thyroxine (T4) hormones which regulate energy consumption in the body and are essential for the growth, development, and maturation of several organs. Iodine is an essential element in the synthesis of T3 and T4 which comes from the diet and is absorbed in the form of iodide. Pendrin, encoded by the *SLC26A4* gene, is responsible for the transport of iodide, hence the importance of this gene in the synthesis of thyroid hormones.

In the KEGG database functional enrichment analyses, the “thyroid hormone synthesis” process was the only significant one. The *SLC26A4* gene, together with *DUOX2* (dual oxidase 2) and *DUOXA2* (dual oxidase maturation factor 2), which belong to the “thyroid hormone synthesis” pathway, were downregulated in the LE group and highly related to each other (Figure 3 and Table S2). The *DUOX2* and *DUOXA2* genes are responsible for the supply of H2O2 peroxide, which acts as an enzymatic cofactor in the iodide oxidation reaction, needed for the synthesis of T3 and T4. Mutations in the *DUOX2* and *DUOXA2* genes have been described in patients with congenital hypothyroidism in humans (Zamproni et al., 2008; Park et al., 2016; Aycan et al., 2017). In another study, Nie et al. (2015) described a SNP in the *DUOX2* gene in the giant panda. This mutation is critical in the synthesis of thyroid hormone, and explains how this carnivorous animal has a specialized diet of bamboo, to which its alimentary tract is poorly adapted. Pandas have exceptionally low energy expenditure, in part due to the reduced size of several vital organs and low physical activity, and because they have circulating levels of the thyroid hormones T4 and T3 that are lower than those expected for a eutherian mammal of comparable size.

The *COLEC12* gene encodes a C-type lectin, known as collectin 12 or CL-P1, that binds to the carbohydrate structures found in invading pathogens. It is responsible for the formation of a receptor that has several functions associated with host defense which promotes the binding and phagocytosis of Gram-positive and Gram-negative bacteria and yeasts. In addition, it also mediates the recognition, internalization, and degradation of oxidatively modified low-density lipoprotein (oxLDL) by vascular endothelial cells, binding to several carbohydrates, including Gal-type ligands, D-galactose, L- and D-fucose (UNIPROT, 2019). In a study conducted with 10,300 Taiwanese individuals, Lin et al. (2017) performed a genome-wide association study (GWAS) related to metabolic syndrome and found an association with the SNP rs16944558 in the *COLEC12* gene, which was also linked to high triglyceride and low HDL cholesterol levels. The authors suggested that this association was due to the fact that *COLEC12* is involved in lipid metabolism, since it plays a role in mediating the uptake of oxidized low-density lipoprotein in vascular endothelial cells.

Another group of genes with reduced expression in the comparison of HE x LE groups was the chemokines *CCL21* and *CXCL17*. CCL21 is considered a constitutively produced homeostatic chemokine, although its production increases during inflammation. This chemokine binds to a cell surface receptor known as CCR7, to exert its function of guiding leukocytes and dendritic cells that express CCR7 to secondary lymphoid organs (Comerford et al., 2013). When CCL21 is not recognized by cells, for example, in CCR7-deficient mice, a delayed and reduced adaptive immune response occurs due to reduced interactions between dendritic cells and T cells in the lymph nodes (Comerford *et al.*, 2013). *CCL21* expression is upregulated in the adipose tissue of cows with moderate negative energy balance during the period of one wee postpartum which requires high energy mobilization (Mellouk et al., 2019). These authors suggested that CCL21 is considered an adipokine, a cytokine secreted by adipose tissue. The chemokine CXCL17 is a small cytokine that belongs to the CXC family and attracts dendritic cells and monocytes (Pisabarro et al., 2006). CXCL17 exhibits an abundant and specific expression pattern in mucosal sites, but the functional significance of its specific expression profile is still undetermined. Studies have documented its ability to recruit immune, anti-inflammatory, and even antibacterial cells, suggesting its role as a homeostatic chemokine. The role of CXCL17 in angiogenesis and tumorigenesis has also been demonstrated in different types of tumors. Still, it is noted that there is not much information about CXCL17, and more specific research should be done to discover its receptor, which is still undetermined, its physiological function, and even its role in tumor immunity (Xiao et al., 2021).

In this present study, the gene ontology analyses highlighted the biological process “negative regulation of leukocyte migration” as the most evident, suggesting that the immune system may contribute in some way to the dynamics of FE. The human liver is regularly not associated with an immune organ, as it is mainly involved in metabolic, nutrient storage, and detoxification activities (Robinson et al., 2016). However, it is now recognized that the liver plays a crucial role as the first defense of the body in the immune system. It houses the largest group of phagocytic cells, which identify and respond to pathogens entering through the digestive tract, as well as internally produced antigens. This is enabled by the advanced ability of the liver to distinguish between self-antigens and foreign substances, such as food antigens or harmful microbes. Acting as an immune-active organ, the liver serves as a protective barrier against external threats, quickly mounting a strong immune response, when necessary, particularly under adverse conditions (Parlar et al., 2023). After appropriate immune activation by pathogen challenge or tissue damage, mechanisms to resolve inflammation are essential to maintain hepatic homeostasis. Failures in these mechanisms can lead to local inflammation, loss of function, and even fibrosis, cirrhosis, and liver failure. An imbalance occurs in an activated liver, as it needs to produce more substances than a liver in a normal state. In a healthy individual, the liver produces a range of serum proteins, including albumin, coagulation factors and complement. During acute infection, hepatocytes are induced to produce a series of antimicrobial proteins, inflammatory mediators, coagulation factors and opsonins, to activate the acute phase response (Robinson *et al.*, 2016). This imbalance may be involved in the loss of FE, since it compromises the metabolic functions of the liver and increases energy expenditure to produce substances of the immune system.

A study by Salleh et al. (2018) provides information about the biological functions of the liver that are potentially involved with FE. Genes also related to the immune system, such as *IFNG* and *IL10RA*, were highlighted as potential candidate genes for the development of new biomarkers of FE. Fonseca et al. (2019) evaluated Nellore bulls for FE using liver samples. Six animals showing high FE and six animals with low FE were collected for protein extraction, digestion and analysis by HPLC-MS/MS. Data were analyzed for differential abundant proteins (DAPs), protein networks and functional enrichment. A total of 42 DAPs were found and the main associated pathways were: microbial metabolism; biosynthesis of fatty acids, amino acids, and vitamins; glycolysis/gluconeogenesis; xenobiotic metabolism and antigen processing and presentation. These publications found association of interferon gamma, interleukins, and antigens presentation which corroborate our results showing that FE is closely related to the functioning of the immune system.

The *FBLN1*, *FBLN2*, and *FBLN7* genes were differentially expressed in this study. They encode fibulin-like extracellular matrix (ECM) proteins. Fibulins are a family of secreted glycoproteins that play an important role in regulating multiple cellular functions, such as adhesion, growth, motility, and survival. To date, eight fibulins have been identified, all of which share a conserved fibulin-like C-terminal domain (Chakraborty et al., 2020). The *FBLN1* gene was less expressed in the rumen in a group of low-weight gain beef steers when compared to the high-weight gain group (Kern et al., 2016). A GWAS conducted by Hong et al. (2019), carried out to investigate genetic markers associated with carcass traits in Korean Hanwoo steers, identified two candidate genes associated with backfat thickness and *FBLN2* was one of them. Mukiibi et al. (2019) evaluated the liver transcriptome of Charolais and Kinsella composite beef steers and found that *FBLN2* gene was differentially expressed between the contrasting groups for the metabolic body weight trait. In addition to cell-cell and cell-matrix interactions in physiological processes, *FBLN7* also inhibits the process of angiogenesis or the formation of new blood vessels (De Vega et al., 2014). In the review by Chakraborty *et al.* (2020), the authors report the role of fibulin 7 in the control of angiogenesis, tooth formation, immune response, inhibiting the inflammatory properties of monocytes and macrophages and in some pathologies such as glaucoma and cancer, especially in gliomas.

In addition to the *FBLN1*, *FBLN2*, and *FBLN7* genes, other genes encoding ECM proteins, such as *DPT*, *LUM*, *LAMC2*, *COL1A2*, and *COL16A1,* were differentially expressed. In the GO functional analyses, many processes involving the ECM were enriched, such as “negative regulation of cell motility,” “negative regulation of cell migration,” “extracellular matrix organization,” and “cell adhesion”. Regarding the cellular component, in the gene ontology analyses, five terms were enriched: “fibrillar collagen trimer,” “collagen trimer,” “collagen-containing extracellular matrix,” “extracellular matrix,” and “extracellular space” (Table S2). These five terms are related to each other, since collagen is present in the ECM, which is the supporting structure that surrounds hepatocytes and other components of liver tissue. In normal liver, the ECM existing in the space of Disse is composed of glycoproteins such as fibronectin, fibulin, and laminin, type IV nonfibrogenic collagen, and proteoglycans. These components form a lattice-like matrix that is essential for providing mechanical support as well as molecular signals for the proper arrangement and functioning of liver cells. When there is liver injury, the composition and density of the ECM change. There is a six- to eight-fold increase in the production of ECM components, and nonfibrogenic type IV collagen is replaced by fibrogenic type I and II collagen (Acharya et al., 2021). These results suggest a strong association between the role of the liver ECM and FE. It is likely that the composition and structure of the hepatic ECM interfere with the transit of molecules and cellular communication, thus potentially interfering in the liver functions.

Among the DEGs found in this work, many showed the initial nomenclature LOC which is generally used to designate unidentified locations or specific genomic loci, but without direct assignment to a known gene. Numbers indicate a specific location in the genome, but do not include a specific designation of a gene. There is little information about these LOC sequences in the literature which indicates that more information is needed to highlight the function of these regions in the bovine genome. LOC112448801 and LOC112444174 were less expressed in the LE group. These two regions were identified as long non-coding RNAs (lncRNAs) located on bovine chromosomes 11 and 24, respectively. Chen et al. (2020) found the region where LOC112448801 is located as a possible candidate gene in their dairy milk temperament study. More studies need to be done to understand the true function of these lncRNAs in feed efficiency.

Although the liver tissue is relevant to metabolic and physiological processes related to feed efficiency, other tissues or biological systems may also play important roles in this phenotype, such as adipose tissue, the digestive system, the endocrine system, the mammary gland, and the immune system. Therefore, exclusive analysis of liver tissue may not fully capture the complexity of FE. 

The complete experiment initially involved 29 bovine animals, which were daily monitored for FE throughout 300 days of lactation. A total of five animals in each HE and LE group, showing extreme RFI values of the normal curve, were selected to maximize the contrast between experimental groups and to facilitate the identification of candidate genes for FE. Additional experiments aiming to detect DEG for feed efficiency should be additionally done to help validating our results. 

Although RNA-seq is a valuable technique to identify differentially expressed genes, it is not a direct measure of protein expression. Gene expression and protein expression are not always directly and linearly correlated due to several factors, such as post-transcriptional regulation, mRNA stability, translation efficiency, and the presence of alternative splicing. Therefore, for a more comprehensive understanding of the mechanisms underlying FE, additional studies of protein expression analyses could be performed, such as Western blotting, immunohistochemistry, among others. This could provide a more complete and robust view of the molecular alterations associated with FE in dairy cattle.

In conclusion, the comparison between high and low feed efficient Girolando cows revealed 149 differentially expressed genes in which 41 were upregulated and 108 were downregulated. Based on our results, some DEG stood out as potential candidate genes, such as *DLK1*, *CACNG4*, *SLC2A12*, *SLC26A4*, *DUOX2*, *DUOXA2*, and *COLEC12*. Functional enrichment analyses showed that DEG participate in specific pathways, possibly influencing FE, such as negative regulation of leukocyte migration, regulation of calcium channel activity, negative regulation of cell migration and adhesion, organization of the extracellular matrix (ECM) and thyroid hormone synthesis. In this way, our results suggest the involvement of solute transport processes, ECM composition, and the role of the immune system in FE. These results could help expanding our understanding of the mechanisms related to FE in dairy cattle, contributing to the development of selection strategies aimed at improving the FE trait.

## Supplementary material

The following online material is available for this article:

Table S1 - List of differentially expressed genes in the liver of lactating Girolando F1 cows selected for contrasting feed efficiency phenotypes: high feed efficiency (HE) x low feed efficiency (LE), obtained by RNA-Seq analyses.

Table S2 - Functional enrichment analyses from differentially expressed genes found in the liver of lactating Girolando F1 cows selected for contrasting feed efficiency phenotypes.

## Data Availability

Sequence data was deposited at the Sequence Read Archive of the NCBI under the accession number PRJNA1330216 (https://www.ncbi.nlm.nih.gov/sra/PRJNA1330216) and will be available on 2026-10-01.
