# Supplementary material for: Gene expression analysis for feed efficiency trait in liver tissue of lactating Girolando cows
Source: Genet Mol Biol. 2026 Jan 19;48(4):e20250036. doi: 10.1590/1678-4685-GMB-2025-0036 (PMC12814931; doi:10.1590/1678-4685-GMB-2025-0036)
Supplement: Table S1 [file 1415-4757-GMB-48-04-e20250036-s1.pdf]

Genetics and Molecular Biology
https://doi.org/10.1590/1678-4685-GMB-2025-0036

Supplementary Material to "Gene expression analysis for feed efficiency trait in liver tissue of lactating Girolando cows"

Table S1 - List of differentially expressed genes in the liver of lactating Girolando F1 cows selected for contrasting feed efficiency phenotypes: high feed efficiency group (HE) x low feed efficiency group (LE), obtained by RNA-Seq analyses.

**Genes *up-regulated* HE x LE**				**Genes *down-regulated* HE x LE**			
**Gene Symbol**	**Log$_2$FC**	***p-value***	**p-adj**	**Gene Symbol**	**Log$_2$FC**	***p-value***	**p-adj**
LOC101907335	7,64	4,2E-06	8,1E-03	*LOC112448801*	-8,02	3,1E-05	3,3E-02
LOC112445540	7,24	8,0E-09	6,9E-05	*KLRF2*	-7,03	2,2E-04	1,1E-01
LOC112446473	5,27	7,5E-04	1,9E-01	*LOC100847268*	-5,53	4,0E-03	4,4E-01
LOC112445652	4,87	3,7E-04	1,4E-01	*LOC100300442*	-5,25	3,6E-03	4,3E-01
LOC104974249	4,38	1,4E-03	2,6E-01	*LOC101907929*	-5,23	2,2E-04	1,1E-01
LOC112442667	4,36	2,2E-03	3,3E-01	*LOC107132214*	-5,21	2,7E-03	3,7E-01
LOC112448028	4,25	5,2E-05	4,3E-02	*LOC112444174*	-5,21	1,4E-05	1,8E-02
SLC38A11	3,95	6,3E-05	4,6E-02	*ASCL2*	-5,05	9,4E-04	2,2E-01
LOC100299144	3,77	6,5E-05	4,6E-02	*PENK*	-4,97	7,3E-03	5,6E-01
LOC101906006	3,59	5,2E-03	4,9E-01	*SUSD5*	-4,60	7,8E-04	1,9E-01
LOC104973018	3,54	4,0E-03	4,4E-01	*LOC104970790*	-4,43	4,5E-03	4,6E-01
LOC112447397	3,02	8,7E-03	6,0E-01	*LOC104969409*	-4,03	3,8E-03	4,4E-01
LOC112448029	2,68	8,9E-04	2,1E-01	*FXYD2*	-4,03	4,6E-04	1,6E-01
EPHA5	2,53	2,5E-03	3,5E-01	*KCNG1*	-3,84	1,6E-03	2,9E-01
RAD51AP1	2,42	1,9E-04	9,9E-02	*APOD*	-3,74	7,0E-04	1,9E-01
CARMIL3	2,40	5,6E-03	5,1E-01	*TMC5*	-3,71	1,3E-03	2,4E-01
LOC112444360	2,27	3,7E-03	4,3E-01	*GPR20*	-3,68	4,0E-03	4,4E-01
DLK1	2,26	7,8E-06	1,3E-02	*SLC26A4*	-3,45	7,0E-03	5,6E-01
LOC508646	2,19	4,1E-03	4,4E-01	*FAT3*	-3,38	1,1E-03	2,4E-01
ENPP3	2,09	3,4E-03	4,2E-01	*LHB*	-3,34	5,3E-03	4,9E-01
CNIH3	2,08	1,2E-03	2,4E-01	*LOC618219*	-3,16	7,4E-03	5,7E-01
LOC101904258	1,97	3,2E-03	4,0E-01	*NRIP3*	-2,80	5,4E-04	1,7E-01
ASTN1	1,96	9,9E-04	2,2E-01	*LOC112441847*	-2,77	6,3E-03	5,3E-01
LOC783540	1,80	5,3E-04	1,7E-01	*LDB3*	-2,76	3,8E-03	4,4E-01
LOC101905518	1,79	8,2E-04	2,0E-01	*ANXA8L1*	-2,65	3,3E-03	4,1E-01
SYCP2L	1,74	7,9E-04	1,9E-01	*FBLN7*	-2,65	4,1E-05	3,7E-02
ZNF383-2	1,67	6,1E-04	1,8E-01	*FBLN2*	-2,54	5,8E-08	2,5E-04
CACNG4	1,61	8,1E-08	2,8E-04	*LOC782884*	-2,54	4,0E-03	4,4E-01
LOC619159	1,50	6,0E-04	1,8E-01	*DUOXA2*	-2,52	4,0E-03	4,4E-01
RAPSN	1,42	7,8E-03	5,8E-01	*CCL21*	-2,45	8,5E-07	2,1E-03
BCAT1	1,33	2,2E-03	3,3E-01	*ANGPT4*	-2,45	1,2E-03	2,4E-01
IFT43	1,31	2,9E-04	1,2E-01	*LOC104974739*	-2,42	6,5E-03	5,5E-01
KLHDC7A	1,27	7,2E-03	5,6E-01	*MGAT3*	-2,29	8,5E-05	5,8E-02
ACOD1	1,16	5,6E-03	5,1E-01	*FOXL1*	-2,27	4,3E-03	4,5E-01
AZGP1	1,16	2,2E-03	3,3E-01	*NRIP2*	-2,23	6,2E-04	1,8E-01
CHD5	1,15	3,0E-03	4,0E-01	*C11H2orf40*	-2,23	3,0E-04	1,2E-01
RALGPS1	1,07	4,2E-04	1,5E-01	*SH3TC2*	-2,21	6,1E-04	1,8E-01
LOC618456	1,03	9,2E-04	2,1E-01	*EFS*	-2,21	2,5E-03	3,5E-01
OASL	1,03	5,0E-03	4,8E-01	*PRRX2*	-2,13	2,2E-03	3,3E-01
CDKL1	1,03	2,4E-03	3,5E-01	*LOC104974345*	-2,11	6,1E-12	1,1E-07
LOC107132251	1,00	2,0E-03	3,3E-01	*MARVELD3*	-2,11	3,0E-04	1,2E-01
				FBLN1	-2,06	3,8E-03	4,4E-01
				TFF3	-2,04	2,2E-04	1,1E-01
				GLP1R	-2,02	3,1E-03	4,0E-01
				MMRN1	-2,00	6,2E-03	5,3E-01

Genes *up-regulated* HE x LE				Genes *down-regulated* HE x LE			
**Gene Symbol**	**Log_2FC**	***p-value***	**p-adj**	**Gene Symbol**	**Log_2FC**	***p-value***	**p-adj**
				MAP1B	-1,97	1,7E-03	3,0E-01
				COLEC12	-1,95	1,2E-06	2,5E-03
				CLDN11	-1,95	9,5E-05	6,3E-02
				COX4I2	-1,95	7,6E-03	5,7E-01
				ZBTB7C	-1,95	1,6E-03	2,9E-01
				WNT7B	-1,95	6,5E-04	1,8E-01
				KCNE4	-1,93	9,7E-04	2,2E-01
				GPX2	-1,85	2,2E-03	3,3E-01
				ADCY2	-1,80	5,2E-03	4,9E-01
				MRGPRF	-1,75	4,5E-03	4,6E-01
				MEOX2	-1,73	1,3E-03	2,4E-01
				DUOX2	-1,69	4,1E-03	4,4E-01
				SLC28A3	-1,66	1,4E-04	8,3E-02
				LUM	-1,64	2,9E-03	3,8E-01
				SVEP1	-1,63	4,2E-03	4,5E-01
				FAM69B	-1,60	6,8E-03	5,5E-01
				CYP2S1	-1,60	2,8E-03	3,8E-01
				RRAD	-1,60	5,5E-04	1,7E-01
				C7	-1,59	1,6E-03	2,9E-01
				SLC2A12	-1,59	6,0E-03	5,3E-01
				FRRS1L	-1,59	2,2E-03	3,3E-01
				COL1A2	-1,55	8,1E-03	5,8E-01
				COL16A1	-1,53	2,7E-04	1,2E-01
				NTRK3	-1,52	8,1E-03	5,8E-01
				LAMC2	-1,51	4,3E-04	1,5E-01
				DPT	-1,50	3,7E-05	3,7E-02
				LOC524810	-1,47	6,8E-03	5,5E-01
				MMP7	-1,47	4,1E-08	2,3E-04
				PI16	-1,47	3,2E-03	4,1E-01
				MCAM	-1,46	3,3E-03	4,1E-01
				CPE	-1,45	7,5E-04	1,9E-01
				ABI3BP	-1,44	2,3E-03	3,4E-01
				CELA1	-1,43	6,7E-03	5,5E-01
				MGP	-1,43	9,6E-04	2,2E-01
				DPP10	-1,41	6,8E-03	5,5E-01
				EVC	-1,38	9,3E-03	6,4E-01
				THY1	-1,37	3,8E-03	4,4E-01
				LOC104975607	-1,36	1,2E-03	2,4E-01
				MN1	-1,33	2,0E-03	3,3E-01
				ADAMDEC1	-1,33	9,0E-04	2,1E-01
				NCMAP	-1,32	8,1E-03	5,8E-01
				SLC6A14	-1,31	7,4E-07	2,1E-03
				BICDL2	-1,30	7,4E-03	5,7E-01
				CXCL17	-1,29	1,3E-05	1,8E-02
				DPYSL3	-1,29	2,1E-03	3,3E-01
				FAM81A	-1,29	1,9E-03	3,3E-01
				MMP28	-1,27	2,8E-04	1,2E-01
				ITGA3	-1,26	1,7E-04	9,2E-02
				F3	-1,25	5,1E-03	4,9E-01
				GEM	-1,18	4,7E-03	4,7E-01
				CCL2	-1,16	5,8E-03	5,2E-01
				MAP1A	-1,15	7,6E-03	5,7E-01
				CTNND2	-1,13	5,9E-03	5,2E-01
				DUSP26	-1,12	8,8E-03	6,0E-01
				CLRN2	-1,11	3,9E-05	3,7E-02
				CD34	-1,07	5,4E-03	5,1E-01

Genes *up-regulated* HE x LE				Genes *down-regulated* HE x LE			
**Gene Symbol**	**Log_2FC**	***p-value***	**p-adj**	**Gene Symbol**	**Log_2FC**	***p-value***	**p-adj**
				CA4	-1,06	6,0E-05	4,6E-02
				IGFBP6	-1,05	1,7E-03	2,9E-01
				FAM13C	-1,05	1,3E-03	2,4E-01
				SCTR	-1,03	1,8E-04	9,8E-02
				KRT19	-1,03	6,0E-04	1,8E-01
				ARHGEF25	-1,03	4,4E-03	4,5E-01
				TM4SF20	-1,02	5,3E-05	4,3E-02
