# Supplementary material for: Gene expression analysis for feed efficiency trait in liver tissue of lactating Girolando cows
Source: Genet Mol Biol. 2026 Jan 19;48(4):e20250036. doi: 10.1590/1678-4685-GMB-2025-0036 (PMC12814931; doi:10.1590/1678-4685-GMB-2025-0036)
Supplement: Table S2 [file 1415-4757-GMB-48-04-e20250036-s2.pdf]

Genetics and Molecular Biology
https://doi.org/10.1590/1678-4685-GMB-2025-0036

Supplementary Material to "Gene expression analysis for feed efficiency trait in liver tissue of lactating Girolando cows"

Table S2 - Functional enrichment analyses from differentially expressed genes found in the liver of lactating Girolando F1 cows selected for contrasting feed efficiency phenotypes.

Biological Process (Gene Ontology)

Gene Ontology Term	Description	Count in network[1]	Strenght[2]	*p-value*[3]
IR:0002686	Negative regulation of leukocyte migration	4 de 35	1.32	3.93E-02
IR:2001257	Regulation of cation channel activity	6 de 120	0.96	4.14E-02
IR:2000146	Negative regulation of cell motility	10 de 214	0.93	5.10E-03
IR:0030336	Negative regulation of cell migration	9 de 204	0.91	5.20E-03
IR:0010810	Regulation of cell-substrate adhesion	7 de 159	0.91	2.74E-02
IR:0030198	Extracellular matrix organization	9 de 236	0.85	1.09E-02
IR:1904062	Regulation of cation transmembrane transport	8 de 248	0.77	4.79E-02
IR:2000145	Regulation of cell motility	16 de 766	0.58	8.00E-03
IR:0007155	Cell adhesion	14 de 676	0.58	2.06E-02
IR:0030334	Regulation of cell migration	14 de 722	0.55	3.43E-02
IR:0022008	Neurogenesis	17 de 1034	0.48	3.66E-02
IR:0007399	Nervous system development	22 de 1519	0.42	2.11E-02
IR:0048731	System development	36 de 2880	0.36	5.10E-03
IR:0050789	Regulation of biological process	88 de 11260	0.16	5.10E-03
IR:0050794	Regulation of cellular process	83 de 10613	0.16	5.20E-03
IR:0065007	Biological regulation	91 de 12011	0.14	5.10E-03

Cellular Component (Gene Ontology)

Gene Ontology Term	Description	Count in network[1]	Strenght[2]	*p-value*[3]
IR:0005583	Fibrillar collagen trimer	3 de 11	1.70	1.04E-02
IR:0005581	Collagen trimer	5 de 56	1.21	5.00E-03
IR:0062023	Collagen-containing extracellular matrix	11 de 202	1.00	8.05E-06
IR:0031012	Extracellular matrix	15 de 358	0.89	1.54E-06
IR:0005615	Extracellular space	30 de 1465	0.58	4.26E-07
IR:0005576	Extracellular region	34 de 2064	0.48	1.54E-06
IR:0031226	Intrinsic component of plasma membrane	20 de 1332	0.44	8.10E-03
IR:0005887	Integral component of plasma membrane	18 de 1271	0.42	3.10E-02

IR:0071944	Cell periphery	58 de 5694	0.27	3.05E-05

KEGG Pathways

Pathway	Description	Count in network[1]	Strenght[2]	*p-value*[3]
bta04918	Thyroid hormone synthesis	5 of 71	1.11	1.93E-02

Subcellular localization (Compartmentes)

Compartiment	Description	Count in network[1]	Strenght[2]	*p-value*[3]
GOCC:0005583	Fibrillar collagen trimer	3 of 11	1.7	2.58E-02
GOCC:0062023	Collagen-containing extracellular matrix	7 of 143	0.95	1.13E-02
GOCC:0031012	Collagen-containing extracellular matrix	9 of 209	0.9	2.80E-03
GOCC:0099512	Supramolecular fiber	10 of 424	0.64	3.48E-02
GOCC:0005615	Extracellular space	12 of 556	0.6	2.58E-02
GOCC:0005576	Extracellular region	31 of 1519	0.57	2.36E-07

[1] The first number indicates how many proteins in your network are annotated with a particular term and the second number indicates how many proteins in total have this term assigned. [2] Log10(observed / expected). this measure describes how large the enrichment effect is. [3] Shown are p-values corrected for multiple testing within each category using the Benjamini–Hochberg procedure.
